# State-level hospital compliance with and performance in the Centers for Medicaid & Medicare Services’ Early Management Severe Sepsis and Septic Shock Bundle

**DOI:** 10.1186/s13054-019-2382-0

**Published:** 2019-03-18

**Authors:** Jordan A. Kempker, Michael R. Kramer, Lance A. Waller, Henry E. Wang, Greg S. Martin

**Affiliations:** 1 Division of Pulmonary, Allergy, Critical Care and Sleep Medicine, Emory University School of Medicine, 49 Jesse Hill Jr Dr SE, Atlanta, GA 30303 USA; 2 Department of Epidemiology, Rollins School of Public Health, Emory University, Atlanta, GA USA; 3 Department of Biostatistics and Bioinformatics, Rollins School of Public Health, Emory University, Atlanta, GA USA; 4 Department of Emergency Medicine, University of Texas Health Science Center at Houston, Houston, TX USA

A recent article by Barbash et al. reported on the first publically available, 2017 data of United States (US) hospital performance on the Centers for Medicare & Medicaid Services (CMS) “Early Management Bundle for Severe Sepsis/Septic Shock” (SEP-1) quality measure [1]. They demonstrate that 87% of hospitals reported SEP-1 data, at an average compliance with all elements of the bundle of 49% (standard deviation (SD) 19%). In addition to their demonstrating the hospital characteristics associated with high SEP-1 performance, an aggregated state-level description is an important complimentary analysis given the state-specific sepsis quality mandates and initiatives existing and forthcoming. Specifically, pre-dating SEP-1 and beginning in 2014, New York required hospitals to implement sepsis care protocols. Also at the time of writing, Illinois and New Jersey are adopting similar mandates while Ohio and Wisconsin are adopting sepsis public health initiatives [2–4].

In our analysis, we utilized a different, larger denominator file of the 4793 hospitals in the CMS Hospital General Information dataset, resulting in a lower proportion (63% vs. 87% in Barbash et al.) of national hospitals with complete reporting of SEP-1 from January 1 to December 31, 2017. Despite this difference, we demonstrated the same national hospital SEP-1 performance at a national mean of 50% (SD 19%). Aggregating the data at the level of the 56 states and territories available, the percent of each state’s hospitals that were compliant with SEP-1 reporting requirements ranged from 16% (North Dakota) to 100% (Rhode Island and Virgin Islands), at an average of 63% (SD 9%). This is comparable to the national average but with a standard deviation demonstrating wide state variation in individual state’s hospital reporting compliance. Furthermore, this variation appears geographically clustered, with lower reporting throughout the north-central part of the continental US (Fig. 1). In regard to each state’s average hospital performance in SEP-1 bundle compliance, the states’ mean hospital SEP-1 performance ranged from 9% (Virgin Islands) to 63% (Hawaii) around a state average of 48% (SD 9%), comparable to the national mean of all hospitals but with a narrower standard deviation. In contrast to states’ hospital reporting compliance, states’ mean SEP-1 scores do not appear to visually cluster within the continental US (Fig. 2)

**Fig. 1:**
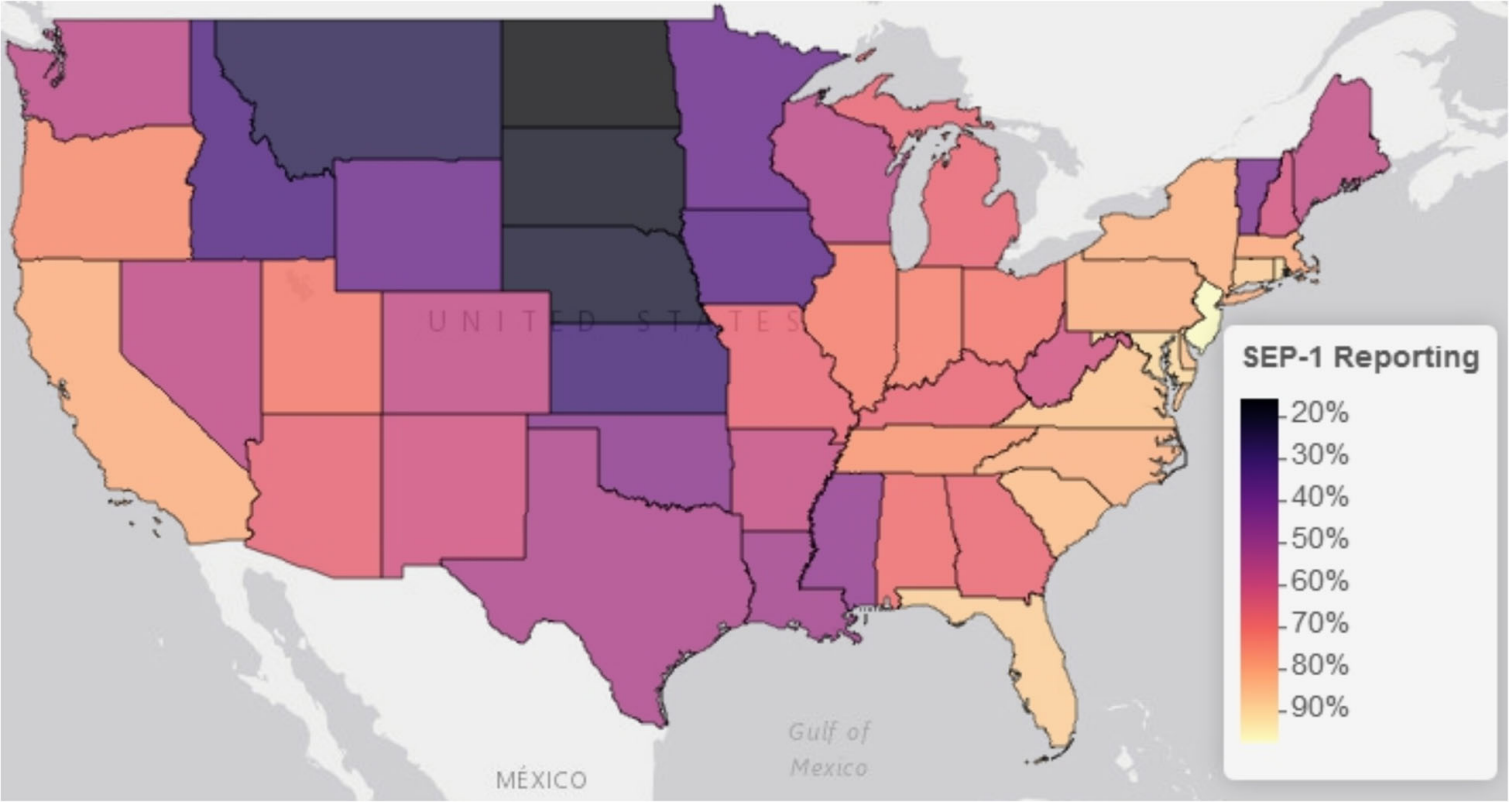
Continental US map of state’s percent of hospitals compliant with SEP-1 reporting. Mapping was performed using the *leaflet* package for R (Version 2.0.1) with the Esri World Gray Canvas basemap (Esri, Delorme, NAVTEQ)

**Fig. 2:**
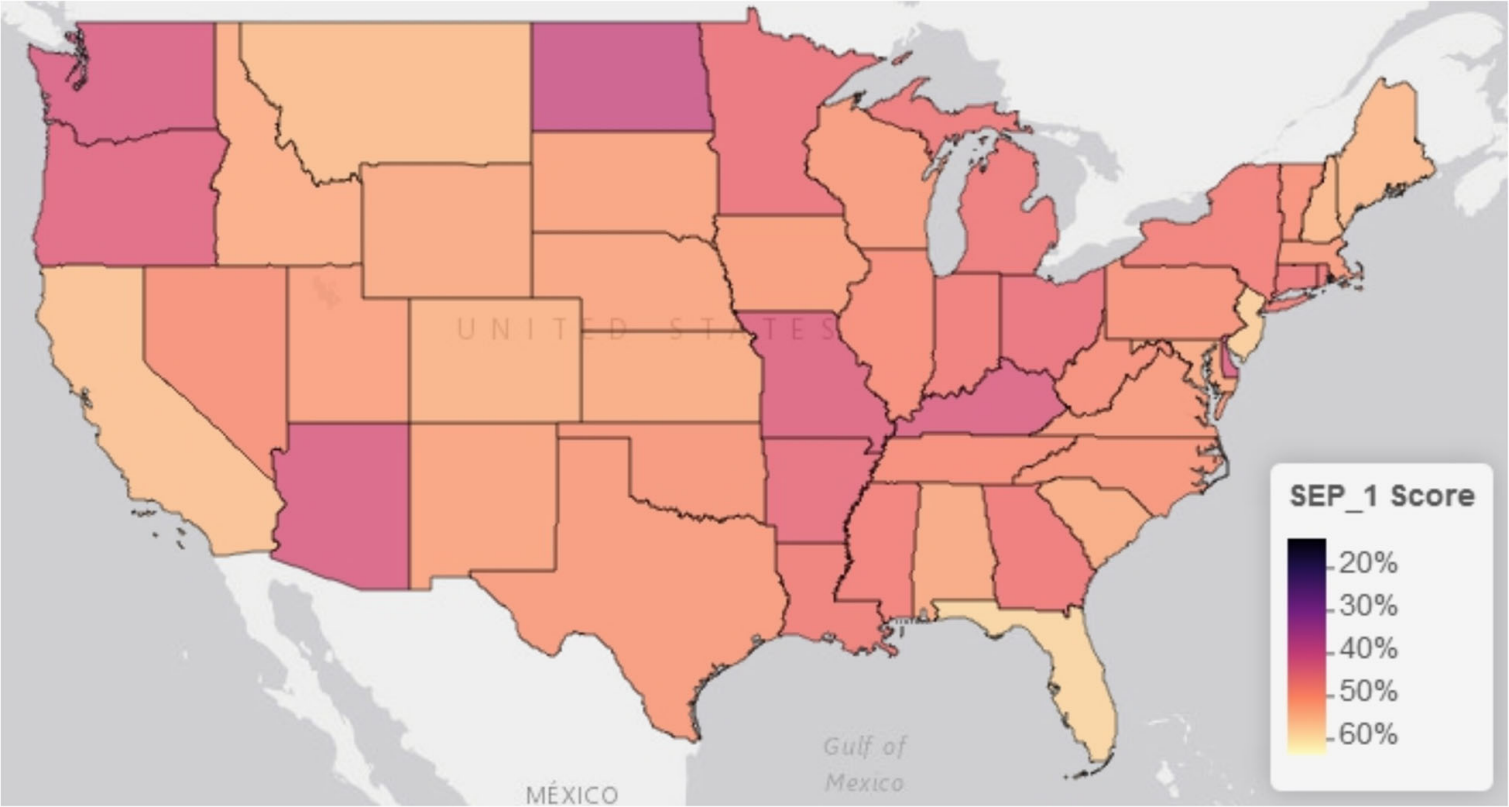
Continental US map of the means of state’s hospitals’ SEP-1 scores. The SEP-1 score represents the percent of patients with sepsis sampled from each hospital that received all components of the Centers for Medicaid & Medicare Services “Early Management Bundle for Severe Sepsis/Septic Shock” (SEP-1) inpatient quality measure. For this figure, hospital scores were summarized as the mean hospital score for each state. Mapping was performed using the *leaflet* package for R (Version 2.0.1) with the Esri World Gray Canvas basemap (Esri, Delorme, NAVTEQ)

These data demonstrate that there is a similar magnitude of variation between states' SEP-1 reporting compliance and performance (SD 9% for both). (Table 1). By the time of this analysis, New York’s hospitals’ reporting compliance with overlapping SEP-1 measure was relatively high, with 82% of hospitals completing SEP-1 reporting. However, New York’s hospitals’ performance in completing the patient-care components of the SEP-1 bundle was just below the national average with 47% (SD 17%) of the state’s hospitals’ sampled SEP-1 patients receiving all components of the SEP− 1 bundle. It remains to be seen whether specific state mandates and initiatives have an impact in addition to the national mandates.

**Table 1:** US state and territories’ hospitals’ reporting compliance and score performance with SEP-1, 2017

State	Hospitals in CMS Universe (*N*)	State’s hospitals reporting SEP-1 data (%)	State’s hospitals with incomplete reporting of SEP-1 data (%)	Hospitals’ SEP-1 score mean (SD)	Hospitals’ SEP-1 score median (IQR)
New Jersey	66	97	3	57.3 (18.3)	58 (44–69)
Rhode Island	11	90.9	9.1	46.2 (21)	39 (33–63)
Maryland	49	89.8	8.2	52.4 (16.4)	51 (40–62)
Florida	184	88	9.8	58.3 (17.9)	58 (47–71)
Washington, DC	8	87.5	12.5	33 (22.8)	30 (21–38)
Connecticut	31	87.1	12.9	45.9 (18.5)	42 (32–59)
Virginia	85	85.9	14.1	50.6 (22.5)	52 (36–66)
Delaware	7	85.7	14.3	42.5 (12.1)	37 (34–49)
South Carolina	60	85	15	52.9 (17.8)	52 (42–66)
North Carolina	105	82.9	17.1	50 (16.8)	48 (30–61)
New York	170	82.4	14.7	47.1 (17.4)	46 (33–60)
California	341	82.1	15.8	55.8 (18.6)	55 (44–69)
Pennsylvania	171	81.9	15.8	49.6 (16.7)	46 (39–58)
Massachusetts	63	81	15.9	50.6 (15.2)	47 (40–63)
Tennessee	108	76.9	18.5	48.7 (16.5)	49 (39–60)
Oregon	60	75	20	43.2 (20.3)	40 (30–61)
Indiana	120	73.3	19.2	46.8 (19.4)	47 (32–60)
Illinois	180	72.8	24.4	48.9 (18.6)	50 (37–60)
Utah	46	71.7	26.1	51.6 (12.9)	51 (43–61)
Ohio	170	71.2	25.3	45 (17.5)	44 (32–59)
Alabama	91	69.2	23.1	52.4 (18.6)	51.5 (39–66)
Georgia	132	67.4	25.8	46.4 (17.1)	47 (34–60)
Michigan	131	67.2	29.8	46.2 (18.2)	43 (33–59)
Kentucky	91	67	31.9	42.2 (16.8)	40.5 (33–49)
Missouri	112	67	30.4	42.8 (22.5)	34.5 (27–57)
Arizona	78	66.7	30.8	42.1 (14.5)	42 (34–49)
New Hampshire	26	61.5	34.6	54.2 (20.8)	57 (42–69)
West Virginia	49	61.2	36.7	49 (19.5)	49.5 (37–65)
New Mexico	41	61	36.6	51.7 (22.7)	46 (32–71)
Arkansas	75	58.7	41.3	44.5 (18.8)	45.5 (33–54)
Maine	33	57.6	42.4	54.6 (19.9)	57 (41–67)
Colorado	80	57.5	38.8	53.8 (16.7)	56 (46–62)
Nevada	35	57.1	42.9	49.6 (17.4)	43 (39–58)
Washington	90	56.7	37.8	42.1 (16.4)	44 (31–54)
Hawaii	23	56.5	43.5	63.2 (13)	64 (53–75)
Wisconsin	126	56.3	39.7	50.7 (16.6)	51 (39–63)
Texas	409	53.5	39.9	50.7 (20.6)	50 (35–65)
Louisiana	119	51.3	42	47 (21)	48 (34–64)
US Virgin Islands	2	50	50	9 (4.2)	9 (8–11)
Mississippi	95	47.4	43.2	47.1 (19.6)	43 (35–55)
Oklahoma	123	46.3	43.1	50.1 (21.3)	48.5 (37–65)
Alaska	22	45.5	50	40 (18)	41 (33–48)
Vermont	14	42.9	57.1	49.3 (12.5)	53 (43–58)
Wyoming	28	39.3	53.6	52.6 (19.3)	46 (39–70)
Minnesota	130	38.5	56.9	45.4 (15)	42.5 (34–59)
Iowa	116	35.3	61.2	51 (19.2)	50 (36–62)
Puerto Rico	52	34.6	63.5	13.3 (20.6)	7 (0–14)
Idaho	42	33.3	66.7	53.2 (17.7)	52.5 (44–66)
Kansas	136	30.9	61.8	52.9 (21.1)	54 (38–67)
Montana	62	25.8	69.4	55 (23.3)	63.5 (40–69)
Nebraska	89	21.3	75.3	51.9 (14.3)	52 (41–60)
South Dakota	58	19	72.4	51.7 (18.6)	56 (38–66)
North Dakota	44	15.9	81.8	39.9 (26.3)	34 (20–52)
American Samoa	1	0	100	NA	NA
Guam	2	0	100	10 (NA)	10 (NA)
Marianna Islands	1	0	100	NA	NA

## Abbreviations

CMS: Centers for Medicaid & Medicare Services
SD: Standard deviation
SEP-1: “Early Management Bundle for Severe Sepsis/Septic Shock” sepsis quality care bundle
US: United States of America
